# Antinociceptive antibiotics-loaded into solid lipid nanoparticles of prolonged release: Measuring pharmacological efficiency and time span on chronic monoarthritis rats

**DOI:** 10.1371/journal.pone.0187473

**Published:** 2018-04-12

**Authors:** Carlos Valdes, Gonzalo Bustos, Jose L. Martinez, Claudio Laurido

**Affiliations:** 1 Laboratory of Neurobiology, Department of Biology, Faculty of Chemistry and Biology, University of Santiago of Chile, Santiago, Chile; 2 Vicerrectory of Research, Development and Innovation, University of Santiago of Chile, Santiago, Chile; Brandeis University, UNITED STATES

## Abstract

Pain is a sensory experience of a complex physiological nature in which is not only involved the nervous system. Among its many features is the development of chronic pain that is more complicated to treat because of the central somatization processes involved, becoming inefficient treatments used in other forms of pain. Among them is the role of glial cells, whose participation is such that some authors have proposed to chronic pain as a gliopathy. Because of this, the drug target of possible treatments focuses on modulating nociceptive response affecting transduction into the central nervous system through affecting synapses in the dorsal horn of the spinal cord. Solid lipid nanoparticles enter the central nervous system, protecting the drug, and in addition to the advantage of having greater absorption surface, all factors that improve drug activity. This work is based on the development and characterization of lipid nanoparticles of solid phase incorporating two antibiotics, minocycline, and ciprofloxacin with antinociceptive properties and challenged them with a rat monoarthritis model using Sprague-Dawley adult male rats. The solid lipid nanoparticles were prepared to modify the lipid, and surfactant amounts to obtain the best encapsulation capacity of the antibiotics, size and z potential. By using the Randall-Selitto test, we measured its pharmacological efficiency as an anti-inflammatory and measuring the time span the antibiotics are active. The encapsulated antibiotics were at least 50% more efficient than the antibiotic alone, and that is possible to measure anti-inflammatory activity up to seven days after the antibiotic application. The former is important for example, in the veterinary field, since a single application of the antibiotic will be necessary for the complete treatment, avoiding excessive stress for the animals. We can conclude that antinociceptive antibiotics encapsulation is a very effective, environmentally safe and inexpensive method for improving the pharmacological efficiency and time span the antibiotics are acting. Since these antibiotics are both anti-microbial and antinociceptive, his use in the field of veterinary presents the advantage of being adequate in single doses, with the saving of time and stress to the animals under treatment.

## Introduction

### Pain pathways

Pain is defined by the IASP as "an unpleasant sensory and emotional experience associated with tissue damage, actual or potential, or described in terms of such damage." From this definition, pain can be understood as an experience that is subjective. For this reason, to differentiate the physiological process of that experience, the term nociception was coined. However, both terms could be considered synonyms understanding the nuances of their meanings. Nociception has a neurobiological origin transported by two pathways, delta, and C fibers. The former possess myelinated axons by which they are classified as fast and C fibers, demyelinated axons that convert them into slow fibers. However, the latter are the primary nociceptive receiving fibers. These terminals have this capacity due to the nociceptors, which are receptors capable of reacting to nociceptive stimuli, whether caused by temperature, mechanical or chemical damage. However, these receptors may be silenced or unresponsive until sensitized. The neurons that make up these fibers are heterogeneous in their sensitivity to stimulation. In turn, these fibers can be classified as mechano-sensitive, mechano-heat-sensitive, and polymodal. The neurons of these fibers perform synapses in the dorsal horn of the spinal cord, where these stimuli can follow three pathways, spino thalamic, espino reticular and espino mesencephalic.

Because of the complexity of these pathways, the first synapse in the dorsal horn of the spinal cord is the critical point to modulate the pain before it ascends the rest of the afferent pathway [1, 2].

### Chronic pain and central sensitization

When tissue or nerve damage occurs, these damaged tissues may remain structures that continue to send signals frequently. These produce a kind of ectopic pacemaker that leads to over-stimulation of some nerve fibers in addition to the activation of new nerve fibers, a phenomenon known as wind up [3], which is nothing more than a successive potentiation after stimulation of the C fibers. This continuous stimulation generates an amplified response that results in changes at the molecular level initiated by the activation of the NMDA receptors of dorsal horn neurons of the spinal cord, which in turn generates a flow of calcium in these postsynaptic neurons. This flow of calcium initiates a cascade of biochemical events involving changes not only at the peripheral level but also affecting the central nervous system. These effects on the central nervous system produce two particular characteristics of chronic pain, allodynia, which is a sharp response to a previously non-painful stimulus and hyperalgesia, which is an increase in nociceptive response to the same painful stimulus. The process of central sensitization is transient and disappears shortly after the stimulus that originates, but as was mentioned above to follow the stimulation this process becomes chronic.

### Role of glia in chronic pain

Both microglia and stimulated astrocytes interact by modulating the synapse in a proposed model as a tripartite synapse [4]. In this model, glial cells actively influence the interaction of neurons. Microglia, for example, releases pro-inflammatory cytokines and BDNF growth factor. On the other hand, astrocytes release interleukin 1β in addition to other factors such as ATP and glutamate. The latter is a ligand of the NMDA receptors, which as mentioned above, generate the intracellular calcium flux, involved in the process of central sensitization.

### Solid lipid nanoparticles

Nanoparticulate drug delivery systems have been particularly interesting because of their properties. Some of the benefits of these systems are, for example, the protection of the drug from its degradation. Due to its small size, it favors its distribution and the contact surface which translates into less amount of active principle for each dose and fewer side effects.

Among the many types of existing nanoparticles, the groups of lipid-based nano compounds are attractive at the technical level for their relative ease of manufacture, low toxicity, and scalable production. Other factors to consider are its good distribution, increased half-life by protecting the compounds in a lipid matrix, having the additional advantage of being able to encapsulate and improve the distribution of classified drugs class II and IV (both of low solubility), According to the BCS (Biopharmaceutics Classification System).

### Antibiotics with antinociceptive and anti-inflammatory properties

We utilized two antibiotics. Ciprofloxacin is a broad spectrum antimicrobial agent, acting on both Gram-negative and Gram-positive pathogens [5]. Also, this antibiotic present antinociceptive activity, even modest due to the route of intraperitoneal administration because the blood-brain barrier permeability of ciprofloxacin was shown to be poor [6] Furthermore, this antibiotic presents also a significant anti-inflammatory effect [7] decreasing IL-1 and TNF-α production. Minocycline is a broad-spectrum tetracycline antibiotic and also possess anti-inflammatory properties. His mechanism of action consists of inhibiting p38 mitogen-activated protein kinases (p38MAPKs) also by inhibiting the activity of metalloproteinase (MMP-9) [8].

## Material and methods

### Drugs

Minocycline was a gift from (Laboratorio Chile, Chile). Salts, ciprofloxacin, stearic acid, tween-80, Poloxamer 188 are from (Sigma-Aldrich, USA). Compritol 888 was a generous gift from (Grupo Mathiesen, Chile), distilled and deionized water was prepared in a Milli-Q water system (Emd-milli-Pore, USA).

### Solid lipid nanoparticles preparation and characterization

SLN nanoparticles were made by the method described by [9] with some modifications. Essentially, the SLN nanoparticles are prepared by heating two 1.5 mL Eppendorf tubes at 85°C, one containing the lipid phase and the antibiotic. The other contains the surfactant. Then, they are mixed and homogenized first with a Tissue Tearor™, model 985370, (Biospec Products, Inc., USA), at 20,000 rpm for 30 sec. Then the mixture is submitted to sonication at 130 W for 90 sec (mrc Sonic-650W, mrclab, Israel). Finally, the prepared nanoparticles are cooled at room temperature (25 ± 5°C).

### Encapsulation capacity

The encapsulation capacity (EC) of the prepared nanoparticles was determined by measuring the amount of free antibiotic present in the suspension media after the nanoparticles preparation. Briefly, the amount X of free drug (X _free drug_) was calculating by diluting the nanoparticles suspension with ethanol (in a variable proportion, depending on the amount of drug present in the nanoparticle suspension). Then, the solution was centrifuged at 10,000 rpm for 30 minutes using a Hettich centrifuge with a 1420-B rotor (Hettich centrifuge, Universal 320R; Andrea Hettich GmbH & Co. KG, Germany). The free drug concentration was measured by using UV/V spectrophotometry at the following wavelengths: 273 nm for minocycline and 268 nm for ciprofloxacin. The amount of encapsulated drug is the result of the initial drug minus the free drug (X _initial drug–free drug_) according to the formula (1):

EC(%)=((Xinitialdrug‑Xfreedrug)/Xfreedrug)x100 (1)

### Nanoparticles size by Dynamic Light Scattering (DLS) and z potential determination

The size of the nanoparticles was determined with the Zetasizer-NanoZs, Malvern Instruments UK, with a 633 nm He-Ne laser as a light source. The nanoparticles were placed in deionized water, and the measurements were done at 25°C. The particle analysis was performed using the Malvern Software Package. For the z potential, the same instrument was utilized. The z values obtained were a mean of triplicates measurements.

The z potential was calculated using the same instrument was utilized. The z values obtained were a mean of triplicate measurements. Briefly, the electrophoretic mobility was determined in an aqueous medium, and linked by the Henry Eq (2):

μE=2εzf(kα)/3ηwhere,μE=electrophoreticmobilityη=dielectricconstantofthesolventz=zetapotentialf(kα)=Henry'sfunctionε=viscosityofsolvent (2)

For larger values of ĸα, Henry’s function approaches 1.5. It is called the Smoluchowski approximation and the relation between those two parameters is direct within this limit.

### Animals

The animals used were rats (Rattus norvegicus) of the Sprague-Dawley line, juvenile males of 125 and 250 g. The animals were obtained from the University of Chile vivarium and kept under a light-dark cycle of 12/12 hours, starting at 8 AM and with food and water *ad libitum*. They were translated in aerated boxes in an air conditioned vehicle and used 24 hours later in order to let the animals acclimatize to the University of Santiago animal facility. All the protocols involving animals were approved by the Ethics Committee of the University of Santiago de Chile (Document number 735, November 28, 2016), and also, according to the Ethical Guidelines for Investigations of Experimental Pain in Conscious Animals [10].

### Intrathecal injections

Intrathecal injections of antibiotics (in free form or nano-encapsulated) were performed by inserting a low caliber needle (25–30g) at the level of the L5-L6 lumbar vertebrae, according to the method proposed by Mestre et al., injecting a maximum volume of 20 μL, both in normal and monoarthritis rats. At the time of intrathecal injections, rats are slightly sedated by the application of gas anesthesia consisting of 2% isofluorane and 98% oxygen, awaiting the complete recovery of the animal to the anesthesia before being subjected to the mechanical pressure analgesia test which is described below [11].

### Monoarthritis induction

The monoarthritis induction was done by injecting 100 μl of Freund's complete adjuvant into his lower right extremity at the tibiotarsal joint to young rats (125 grams). This adjuvant is composed of mineral oil and other excipients. The so-called complete is due to possessing Mycobacterium butiricum bacterial detritus. This solution generates an inflammation in the limb that begins 14 days after the Freund’s adjuvant injection. A third week is expected with the inflammation to be able for working with these animals since after this time the pain is considered chronic. Then, at day 15 after the monoarthritis induction, the rats were divided into the following categories and submitted to the protocol of injections as described in the following Table 1:

**Table 1:** In day 15 after the monoarthritis induction, the rats were divided into the following categories and submitted to the protocol of injections.

Ciprofloxacin intrathecal injections	Abbreviation	Dosage μg /rat at day 15.
Free in normal rats	Cipro (N)	20
Free in monoarthritic rats	Cipro (M)	20
Loaded into SLN in normal rats	Cipro nano (N)	20
Loaded into SLN in monoarthritic rats	Cipro nano (M)	10
Saline in normal rats	Saline (N)	20
Saline in monoarthritic rats	Saline (M)	20
Minocycline intrathecal injections	Abbreviation	Dosage μg /rat at day 15.
Free in normal rats	Mino (N)	20
Free in monoarthritic rats	Mino (M)	20
Loaded into SLN in normal rats	Mino nano (N)	20
Loaded into SLN in monoarthritic rats	Mino nano (M)	10
Saline in normal rats	Saline (N)	20
Saline in monoarthritic rats	Saline (M)	20

Then, we measured the antinociceptive effect by using the Randall-Selitto test at days 15, 17, 19 and 21.

### Randall-Selitto test

This test is known as Randall's test [12]. It is an algesiometric test of mechanical nociception. Briefly, the rats are left in a particular room with dim light and a temperature of 25°C for 30 minutes before the experiment to reduce the stress and anxiety of reaching a new place. After that time, the animal is held with one hand by an operator who inserts one of its lower extremities into the team's pliers. The animal leg is maintained in the indicated area without forcing it, gradually applying pressure until the animal feels discomfort and removes the tip of the pincer and/or vocalizes. The applied pressure level is recorded by the equipment in a dimensionless unit which can be converted into grams using a conversion factor.

### Animal protocol

In total, 76 rats were utilized in the present study, both normal and monoarthritis rats. The animals under study were divided into the following categories, see Table 2:

**Table 2:** Summary of the whole animals utilized during the developing of this work.

	Minocycline		Ciprofloxacin		Number
Animals	Free	Encapsulated	Free	Encapsulated	
Normal rats, 250 g weight.	12	6	12	6	36
Normal rats, 250 g weight.	2		2		4
Monoarthritic rats 125 g weight.	12	6	12	6	36
Total					76

### Identification of the antibiotics injected intrathecally by High Performance Liquid Chromatography (HPLC)

In order to confirm the presence of the antibiotics in the cerebrospinal fluid (CSF) after the intrathecal injections of the antibiotics, a HPLC was run with the CSF extracted from the rats. The CSF was extracted by using a device developed in our Lab [13]. Briefly, the rat is anesthetized with a short isofluorane application (2.0% in 98% oxygen) and placed in a stereotaxic system in which the rat ears are secured by rods. Then the head is positioned downwards at an angle of 45° from horizontal, then inserting a needle into the cisterna magna using a descender device. Finally, a vacuum system withdraws the CSF. The CSF extracted was analyzed by HPLC using a mixture between two mobile phases. The phase A consisted of a solution of KH_2_PO_4_ 0.025 M adjusted to pH 3. The phase B formed by acetonitrile under a gradient starting in 5% of B until reaching 60%, with a total time of 15 minutes per sample. The equipment utilized was a HPLC Waters 600 PAD 2996, provided with a column Waters Symmetry C18 (3.9 x 150 mm)

### Visualization of the nanoparticles with Scanning Electronic Microscope (SEM)

The equipment utilized for the visualization of the nanoparticles was a Zeiss, model EVO MA 10 with an EDS Penta FET Precision detector, Oxford Instruments X-act. For the sample preparation, three 3 μL of nanoparticles solution were placed in the grid diluted 10, 50 and 100 times. After air drying overnight, the nanoparticles were covered with a gold layer of approximately 15 nm thickness. The later using a Cressington 108 Sputter Coater. The pictures were obtained at 15 KV amplification.

### In vitro release of minocycline and ciprofloxacin antibiotics

In vitro release of antibiotics was done in triplicate. At time zero 21 tubes containing 1 ml of the nanoparticles solution for minocycline and 21 tubes for ciproflozaxin were submitted to orbital agitation at 350 RPM. Starting at day zero, three tubes of each minocycline and ciprofloxacin were measured spectrophotometrically and the antibiotic concentration was determined. The same procedure for the remaining tubes each day up to day seven. Then, the amount of antibiotic released each day is calculated as the mean of the three tube measurements for each day, subtracting the amount calculated the previous day and expressed as percentage of the remaining encapsulated antibiotic.

### Statistical analysis

Results were expressed as means ± standard error of the mean (SEM). All the statistical analysis was done using two-way ANOVA, with a p< 0.01 being significant. The calculations were done with the SigmaPlot, v12 for Windows (2011, ®Systat Software, Inc.)

## Results

With the purpose of using the most effective nanoparticles for encapsulating the antibiotics, the nanoparticles were prepared with different formulations, varying the lipid and emulsifier composition in various proportions as shown in Table 3.

**Table 3:** Surfactants and proportions utilized in the manufacture of the nanoparticles.

Nanoparticles formulation	Composition
Compritol	100%
F 8:2	80% Compritol, 20% Stearic acid
F 6:4	60% Compritol, 40% Stearic acid
F 5:5	50% Compritol, 50% Stearic acid
F 4:6	40% Compritol, 60% Stearic acid
F 2:8	20% Compritol, 80% Stearic acid
Stearic acid	100%

Table shows the different surfactants and proportions utilized in the manufacture of the nanoparticles.

After testing the EC for all the different formulations, the F 5:5 for minocycline and F 2:8 for ciprofloxacin produced the best EC. When compared the encapsulation efficiency between poloxamer and tween 80, there was a significative difference between both, being for minocycline p = 0.0008 and for ciprofloxacine p = 0.0002.

### Size and z potential determinations

Every formulation was tested in their EC, and the highest were used. In this case, the best formulations are described in Table 4 (S1 Table):

**Table 4:** Size and z potential determinations for nanoparticles formulation.

Nanoparticles formulation	Size (nm)	EC (%) Poloxamer	z potencial (mV)
Minocycline F 5:5	113 ± 8.3	66.4 ± 5.9	-1,3 ± 0.2
Ciprofloxacin F 2:8	398 ± 6.4	84.3 ± 5.6	-12,5 ± 0.5
Empty	77 ± 11	-	-

Table shows the highest EC together with the nanoparticles size and z potential. Values are mean ± standard error of the mean (S1 Table).

**Fig 1:**
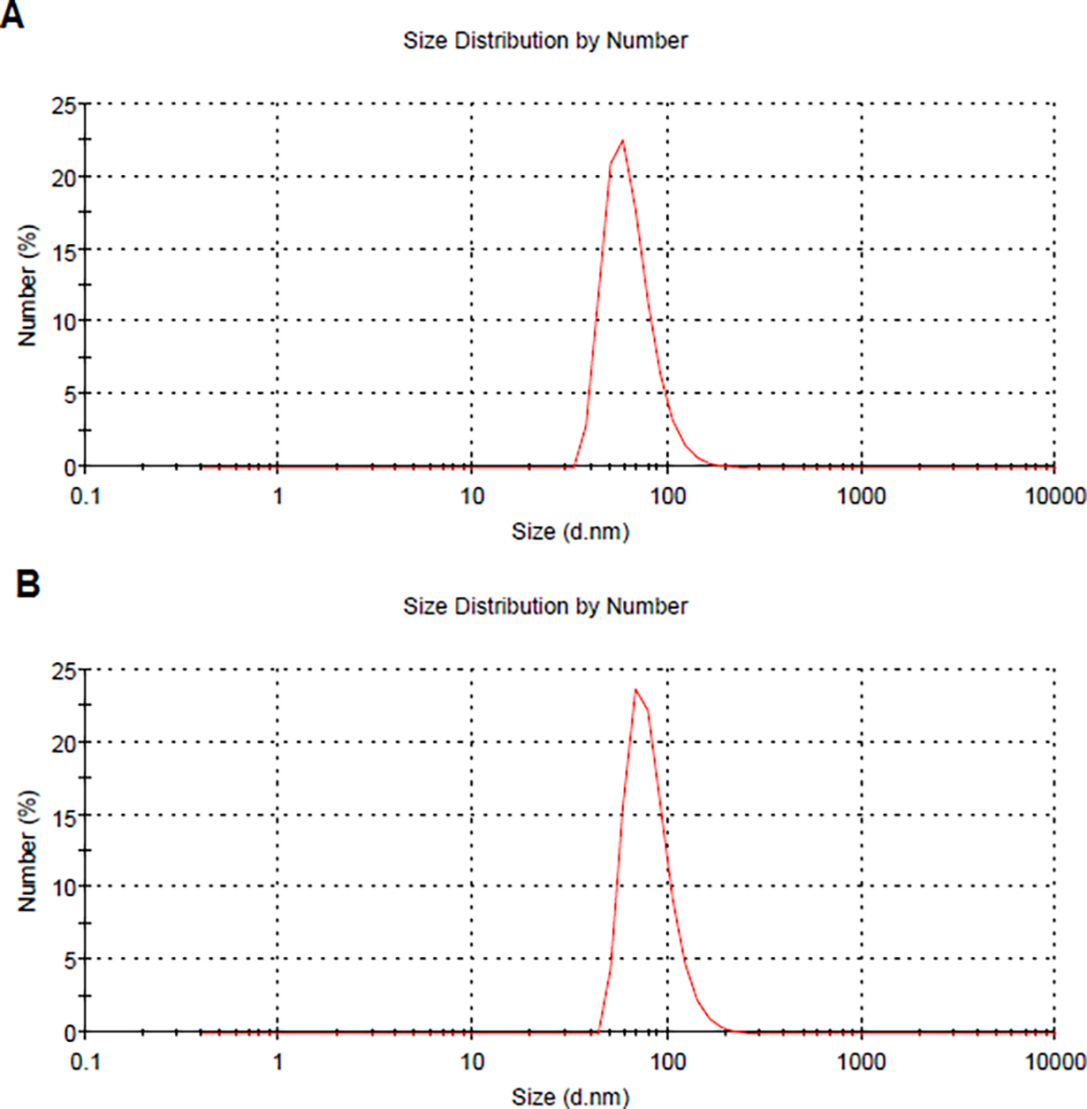
Figure shows the size distribution by DLS of a sample of empty nanoparticles. (A) Diameter of the nanoparticles is 64.6 nm, with a width of 20.7 nm. (B) Diameter of the nanoparticle is 81.6 nm, with a width of 24.0 nm.

Fig 1 shows two representative runs for the DLS size distribution determination. They correspond to empty nanoparticles (S1 Fig).

#### HPLC determination of CSF presence of minocycline and ciprofloxacin

**Fig 2:**
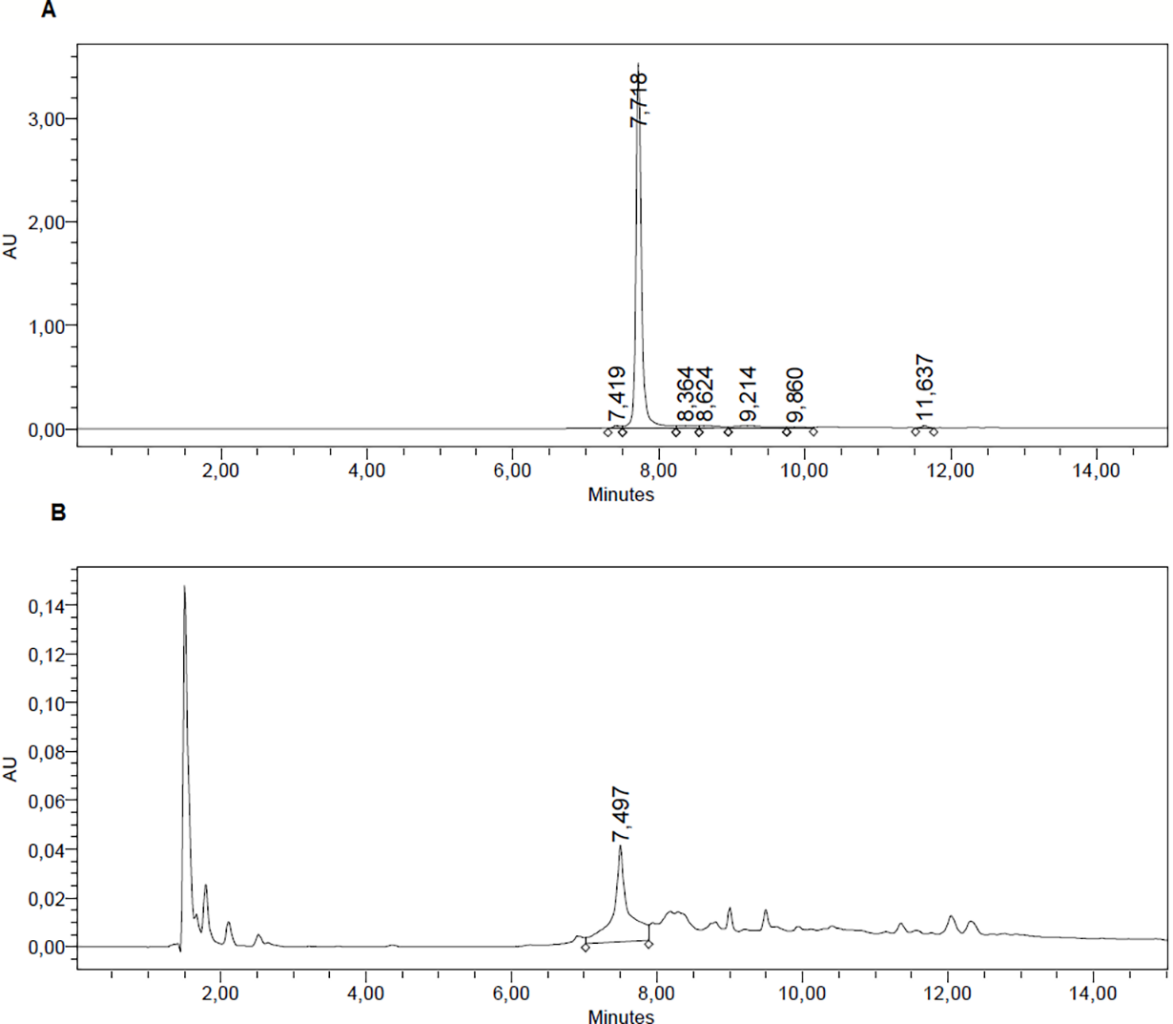
Figure shows the HPLC for minocycline for the pure antibiotic (A). After completion of one experiment, (minocycline injection, Randall-Selitto test) the CSF was withdrawn and measured by HPLC, (B). It can be observed that there was presence of the antibiotic, even though at low concentration in the CSF.

**Fig 3:**
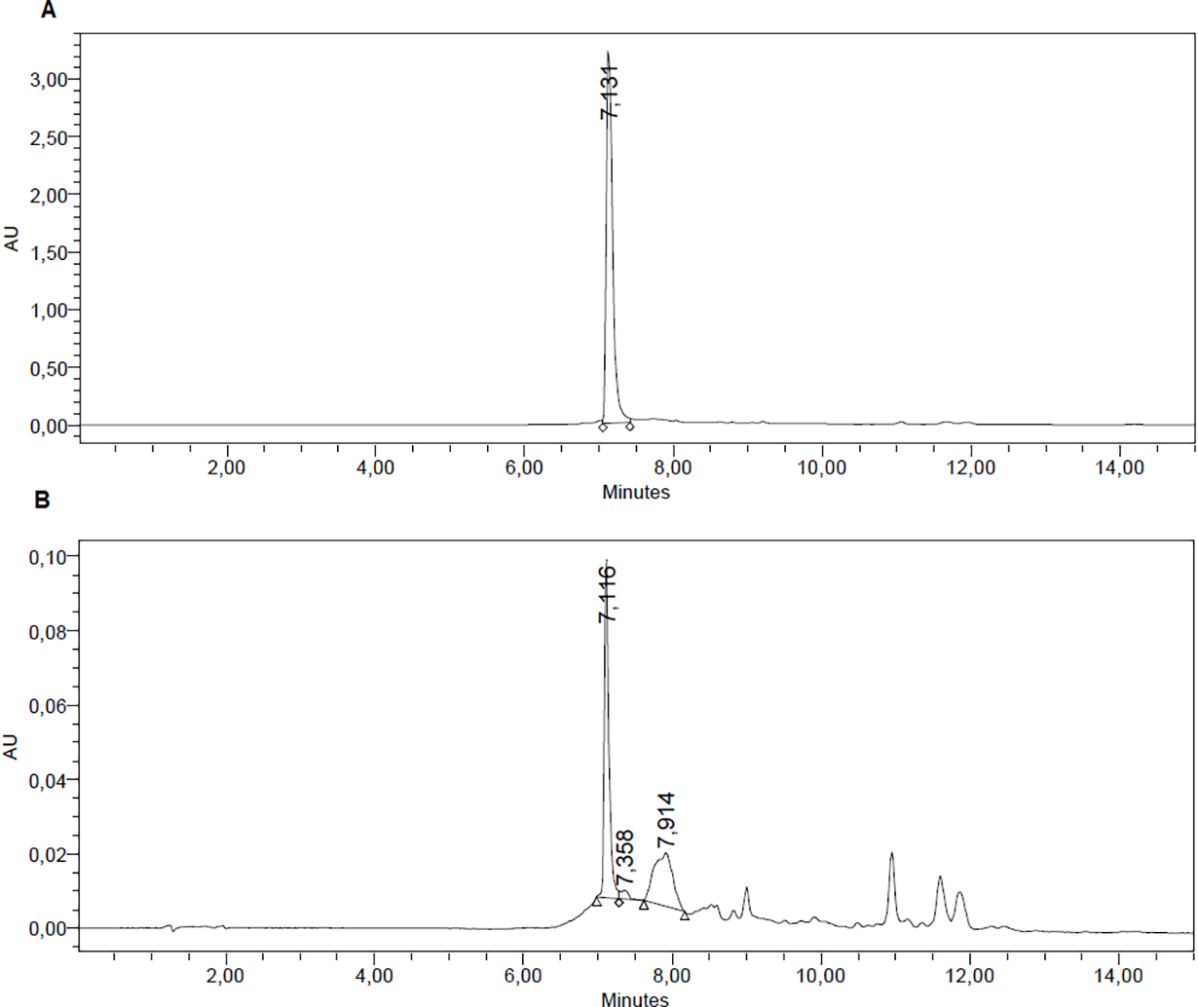
Figure shows the HPLC for ciprofloxacin for the pure antibiotic (A). After completion of one experiment, (minocycline injection, Randall-Selitto test) the CSF was withdrawn and measured by HPLC, (B). It can be observed that there was presence of the antibiotic, even though at low concentration in the CSF.

The presence of both antibiotics was done by HPLC. After one experiment, the CSF was extracted and submitted to HPLC. This constitutes a confirmation of the correct delivery of the antibiotics by intrathecal injection onto the rat CSF. Two runs for minocycline and ciprofloxacin are shown in Figs 2 and 3, respectively (S2 Fig and S3 Fig). These chromatograms are from two additional normal rats injected, submitted to the Randall-Selitto test and then, their CSF was extracted and analyzed. No further experiments were done with these rats, since the volume of CSF extracted is not recuperated from one day to another, making them useless for long-term experiments.

#### Visualization of the nanoparticles with Scanning Electronic Microscope (SEM)

**Fig 4:**
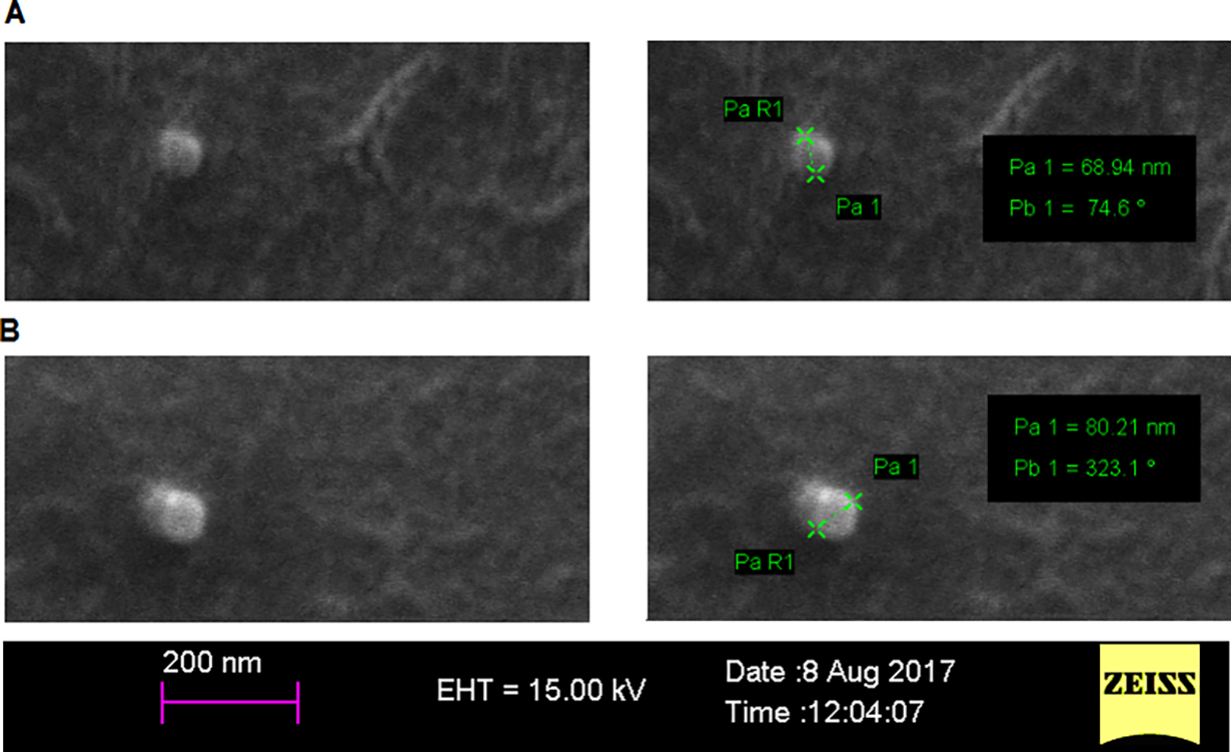
Figure shows SEM pictures of two representative empty nanoparticles. The nanoparticles appear rounded, sometimes aggregated in two, but never agglomerated. (A) Image of the smaller nanoparticles found, and the biggest empty nanoparticles found (B).

Fig 4 shows two pictures of nanoparticles found in two principal sizes. A) shows the smaller nanoparticles found with a diameter of around 69 nm and in B), the larger nanoparticles of 80 nm diameter (S4 Fig).

#### *In vitro* release of encapsulated minocycline and ciprofloxacin

**Fig 5:**
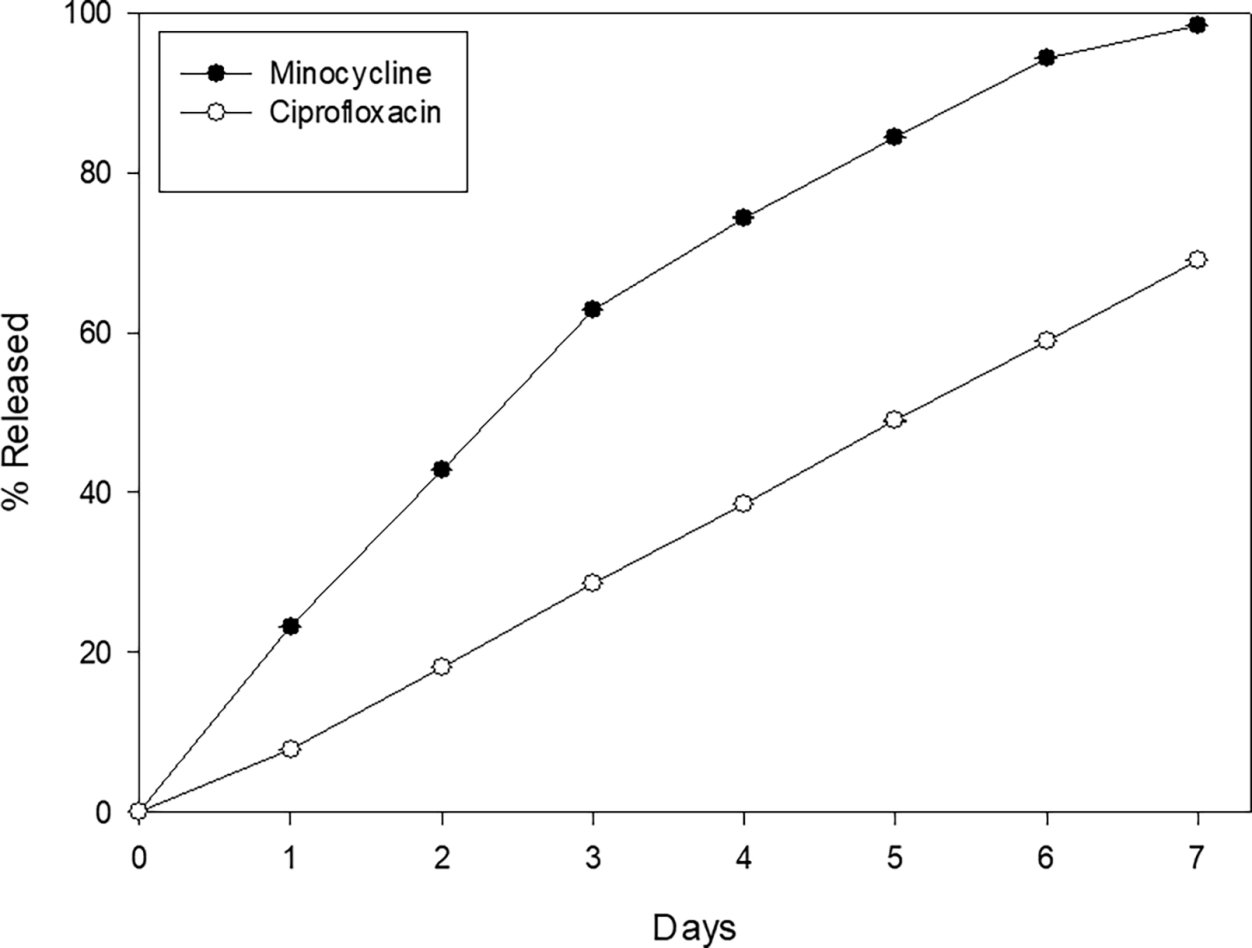
Figure shows the *in vitro* release of both antibiotics over seven days time span. Figure shows the *in vitro* release from nanoparticles of both antibiotics in a time span of seven days. It can be seen that in general, the percentage release of both antibiotics is nearly linear on time, indicating a continuous release. Results are expressed as mean ± SE of the mean.

Fig 5 shows the release profile of minocycline and ciprofloxacin. It can be seen that for both antibiotics (especially for minocycline) there are still antibiotic present at day seven, indicating the slow release of the nanoparticles formulation utilized in this work (S5 Fig).

**Fig 6:**
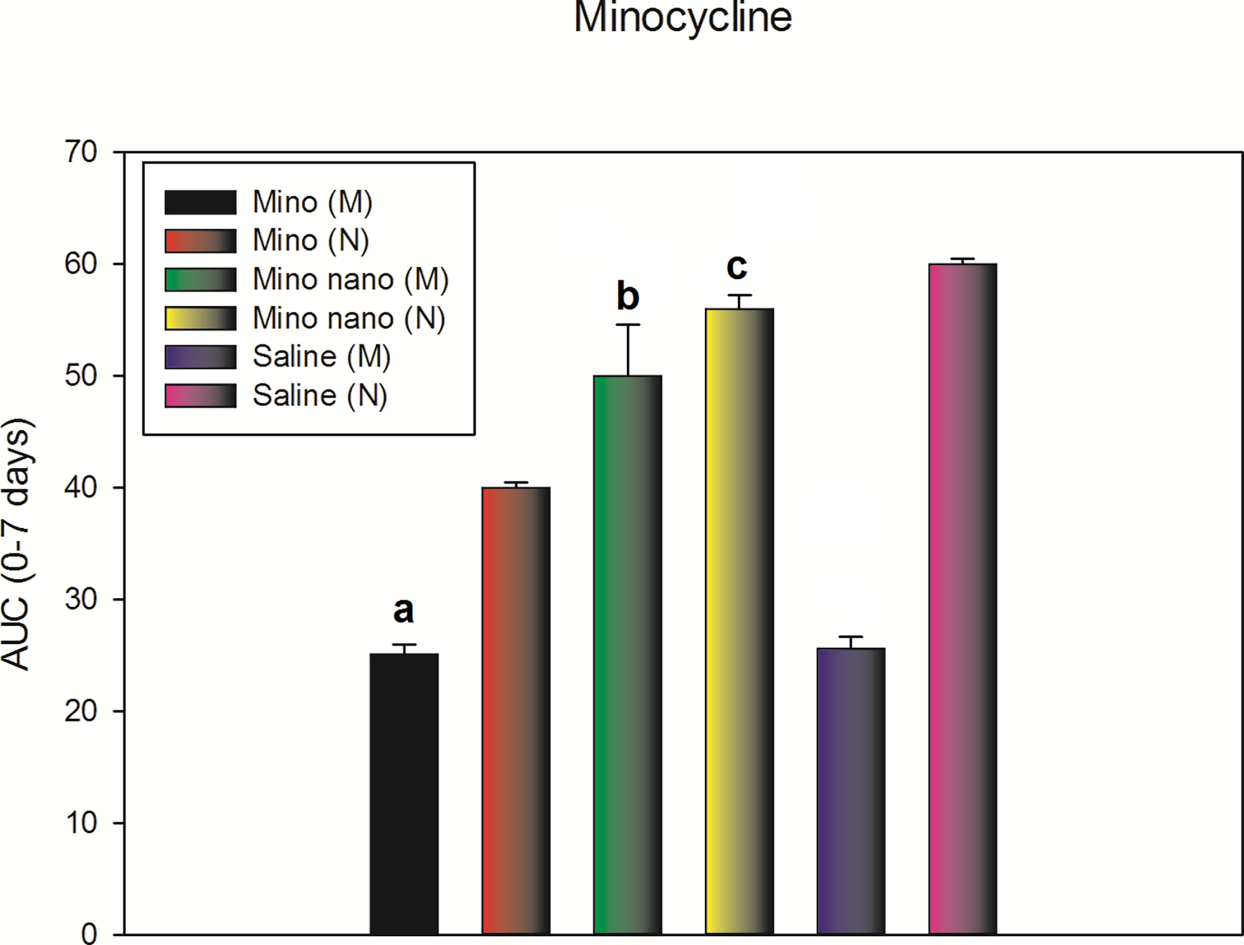
The figure shows the AUC of the intrathecal injections of free minocycline in monoarthritic rats (Mino (M)); intrathecal injections of free minocycline in normal rats (Mino (N)); minocycline encapsulated into SLN in monoarthritic rats (Mino nano (M)); minocycline encapsulated into SLN in normal rats (Mino nano (N)); Monoarthritic rat injected only with artificial CSF (Saline (M)), and normal rats injected only with artificial cerebrospinal fluid (Saline (N)), expressed as the AUC taken from time zero to seven days. (Specifically, day 15 after the induction of monoarthritis, then at day 16 a Randall-Selitto test, followed by day 17, 19 and 21). There were significant differences when comparing “a” with “b" or "c", but not between “b” and “c”, meaning there was a similar antinociceptive effect of the Mino nano (M) rats compared to Mino nano (N) despite the fact the dose was half (10 μg/rat) for Mino nano (M), compared with 20 μg/rat for Mino nano (N). Results are expressed as mean ± SE of the mean. P <0.01 (two-way ANOVA).

**Fig 7:**
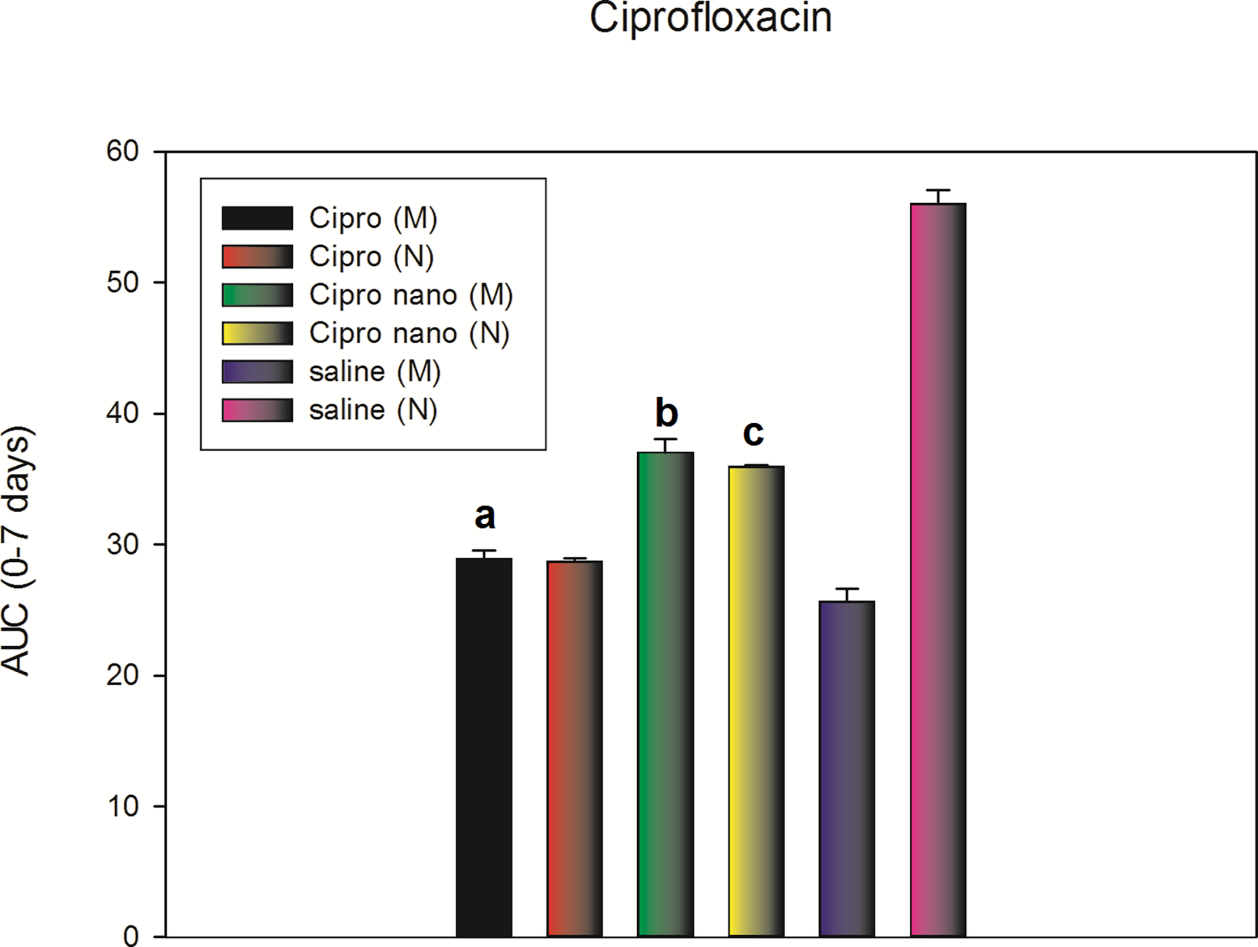
The figure shows the AUC of the intrathecal injections of free ciprofloxacin in monoarthritic rats (Cipro (M)); intrathecal injections of free ciprofloxacin in normal rats (Cipro (N)); ciprofloxacin encapsulated into SLN in monoarthritic rats (Cipro nano (M)); ciprofloxacin encapsulated into SLN in normal rats (Cipro nano (N)); Monoarthritic rat injected only with artificial CSF (Saline (M)), and normal rats injected only with artificial cerebrospinal fluid (Saline (N)), expressed as the AUC taken from time zero to seven days. (Specifically, day 15 after the induction of monoarthritis, then at day 16 a Randall-Selitto test, followed by day 17, 19 and 21). There were significant differences when comparing “a” with “b" or "c", but not between “b” and “c”, meaning there was a similar antinociceptive effect of the Cipro nano (M) rats compared to Cipro nano (N) despite the fact the dose was half (10 μg/rat) for Cipro nano (M), compared with 20 μg/rat for Cipro nano (N). Results are expressed as mean ± SE of the mean. P <0.01 (two-way ANOVA).

Fig 6 and Fig 7. Antinociceptive effect of the intrathecal application of the antibiotics, free or encapsulated in nanoparticles quantified in a time span of 7 days and expressed as the area under the curve (AUC) between days 0 and 7 (S6 Fig and S7 Fig). Shown in the following figures.

There was an antinociception corresponding to an increase in vocalization threshold of around 99% for Mino (M) compared with Mino nano (M). In the other hand, when comparing Mino nano (M) with Mino nano (N), there was no change. This result is interesting since the dose applied for Mino nano (M) was 10 μg/rat compared with Mino nano (N) of 20 μg/rat. For ciprofloxacin, the effect was lower, but following the same tendency of minocycline. There was an antinociception corresponding to an increase in vocalization threshold of around 30% for Cipro (M) compared with Cipro nano (M). In the other hand, when comparing Cipro nano (M) with Cipro nano (N), there was no change. This result is interesting since the dose applied for Cipro nano (M) was 10 μg/rat compared with Cipro nano (N) of 20 μg/rat.

## Discussion

In the search for strategies to treat chronic pain, the use of nanotechnology applied to the field of pharmacology seems to be one of the most promising applications. The information obtained in this work was useful to demonstrate in general how the efficiency of a drug can be improved by improving the form of encapsulation. At a more particular level, it also served to reaffirm the utility of two drugs that although their most described and known activity is that of being antibiotics have been shown to be efficient in their analgesic function. Once the tests with free drugs and encapsulated in nanoparticles can be made, observations can be performed in a better context on the data obtained. According to some authors [14], the particles with values between +10 and -10 mV are considered to be neutral (less than -10 mV). Despite their small size, they could be expected to form larger particles over time similar to liposomes [15]. This increase in volume does not appear to affect the function of this type of particles significantly. Having a negative z potential avoids toxic effects at the cellular level due to the disruptive effect on the biological membranes that positively charged molecules might have. The increase in potential in larger molecules is understood to be proportional to the size of the particle with the number of groups that can confer this potential value. In some works such as that of the group of Hu et al., 2005 [16], the z-potential value is proposed as a measure of stability in colloidal dispersions.

Among the surfactants used the one that achieved a higher percentage of encapsulation was the poloxamer188 which has coherence realizing that its hydrophilic-lipophilic balance index [17] is greater than that of Tween 80 [18] which facilitates better the formation of the emulsion in an aqueous medium emulsion [19].

It is tempting to study the kinetics of SCF antibiotic content by sampling this compartment on time and measured by HPLC. Nevertheless, even though is possible to extract CSF in a low invasive way [13], the availability of this fluid is very low in volume and very difficult to recuperate by the rat metabolism. In our case, we tested this procedure only for making sure that the injected antibiotics were present in the CSF, and a few other experiments at days 3 were done with the injected rat, but not further time experiments.

Regarding the DLS size determination, they compare well with the SEM microphotography reported. These correspond to empty nanoparticles; the antibiotic loaded ones are bigger reflecting the presence of antibiotics in his structure. One problem found was to follow on time the nanoparticles in the CSF. The HPLC method is impractical and after a short time (day 3), the antibiotic signal disappear or falls lower than the resolution of the HPLC determination. Other possibilities can be considered, that the nanoparticles are phagocyted by the glial cells or transferred to by macrophages [20]. The *in vitro* studies for antibiotics release over time showed the presence of antibiotics even at day seven, releasing them in a quasi linear fashion, so at least *in vitro*, the nanoparticles behave as low release system.

No doubt the most relevant information that the animal studies delivered was that nanoparticle-encapsulated drugs showed effects on the *in vivo* tests that equate the responses of the drug administered in free form even though the dose was half of its minimum effective dose [21] as revealed by the AUC (0–7 days) for both antibiotics. It serves as evidence to demonstrate that by encapsulation the drugs improve their efficiency compared to their administration in free form. The former is valid for antibiotics used successfully as antinociceptive drugs.

It could be inferred that this improvement in its efficiency would be due to its encapsulation in nanoparticles, since both in spite of having a similar pharmacological activity, have different chemical natures.

## Conclusion

In conclusion, the nanoencapsulation of minocycline and ciprofloxacin improved the efficiency of both drugs in their antinociceptive role, achieving a 99% of antinociception for minocycline and 30% for ciprofloxacin (S1 Table; S6 Fig and S7 Fig). Also, the time span of the antibiotics activity makes this procedure useful for single doses treatments on farm animals. As mentioned previously, being drugs of different chemical natures and having a similar increase of efficiency in analgesia, it can be affirmed that this incorporation in a lipid matrix with particles in nanometric scale is the main responsible for this improvement [5–8]. Possible future applications could be given the nanoparticles orally because it has been described that SLN administration by oral route to the intestinal mucosa is translocated to the lymphatic system where it is finally able to pass the blood-brain barrier and reach the central nervous system [22]. Thus, this work seeks to be a contribution to the search and development of new tools for the mitigation of pain.

## Supporting information

S1 Table Data of size and z potential determinations for nanoparticles formulation. (XLSX)

S1 Fig Shows the size distribution by DLS of a sample of empty nanoparticles A: Diameter of the nanoparticles is 64.6 nm, with a width of 20.7 nm. B: Diameter of the nanoparticles is 81.6 nm, with a width of 24.0 nm.
(DOCX)

S2 Fig Shows the HPLC for the pure antibiotic (A). After completion of one experiment, (minocycline injection, Randall-Selitto test) the CSF was withdrawn and measured by HPLC, (B).
(DOCX)

S3 Fig Shows the HPLC for ciprofloxacin for the pure antibiotic (A). After completion of one experiment, (minocycline injection, Randall-Selitto test) the CSF was withdrawn and measured by HPLC, (B).
(DOCX)

S4 Fig Shows SEM pictures of two representative empty nanoparticles. The nanoparticles appear rounded, sometimes aggregated in two, but never agglomerated (A). Shows SEM pictures of two representative empty nanoparticles. Image of the smaller nanoparticles found, and the biggest empty nanoparticles found (B).
(DOCX)

S5 Fig Data of in vitro release of both antibiotics over seven days time span. (XLSX)

S6 Fig Shows the AUC of the intrathecal injections of free minocycline in monoarthritic rats (Mino (M)); intrathecal injections of free minocycline in normal rats (Mino (N)); minocycline encapsulated into SLN in monoarthritic rats (Mino nano (M)); minocycline encapsulated into SLN in normal rats (Mino nano (N)); Monoarthritic rat injected only with artificial CSF (Saline (M)), and normal rats injected only with artificial cerebrospinal fluid (Saline (N)), expressed as the AUC taken from time zero to seven days. (XLSX)

S7 Fig The figure shows the AUC of the intrathecal injections of free ciprofloxacin in monoarthritic rats (Cipro (M)); intrathecal injections of free ciprofloxacin in normal rats (Cipro (N)); ciprofloxacin encapsulated into SLN in monoarthritic rats (Cipro nano (M)); ciprofloxacin encapsulated into SLN in normal rats (Cipro nano (N)); Monoarthritic rat injected only with artificial CSF (Saline (M)), and normal rats injected only with artificial cerebrospinal fluid (Saline (N)), expressed as the AUC taken from time zero to seven days. (XLSX)

## Abbreviations

Wind up: Frequency-dependent increase in the excitability of spinal cord neurones, evoked by electrical stimulation of afferent C-fibers
C-fiber: C-fibers are one class of nerve fiber found in the nerves of the somatic afferent sensory system
BDNF: Brain-derived neurotrophic factor
NMDA receptor: N-methyl-D-aspartate receptor
SLN: Solid Lipid Nanoparticles
rpm: revolutions per minute
W: Watts
Freund's complete adjuvant: Freund's adjuvant is a solution of antigen (Mycobacterium butiricum walls) emulsified in mineral oil and used as an immunopotentiator
Tween 80: Laboratory detergent
Poloxamer: hydrophilic non-ionic surfactant
O / W emulsion: Oil / Water Emulsion
