# Supplementary material for: Antinociceptive antibiotics-loaded into solid lipid nanoparticles of prolonged release: Measuring pharmacological efficiency and time span on chronic monoarthritis rats
Source: PLoS One. 2018 Apr 12;13(4):e0187473. doi: 10.1371/journal.pone.0187473 (PMC5896893; doi:10.1371/journal.pone.0187473)
Supplement: S1 Fig — Shows the size distribution by DLS of a sample of empty nanoparticles A: Diameter of the nanoparticles is 64.6 nm, with a width of 20.7 nm. B: Diameter of the nanoparticles is 81.6 nm, with a width of 24.0 nm. (DOCX) [file pone.0187473.s002.docx]

**Fig 1A**


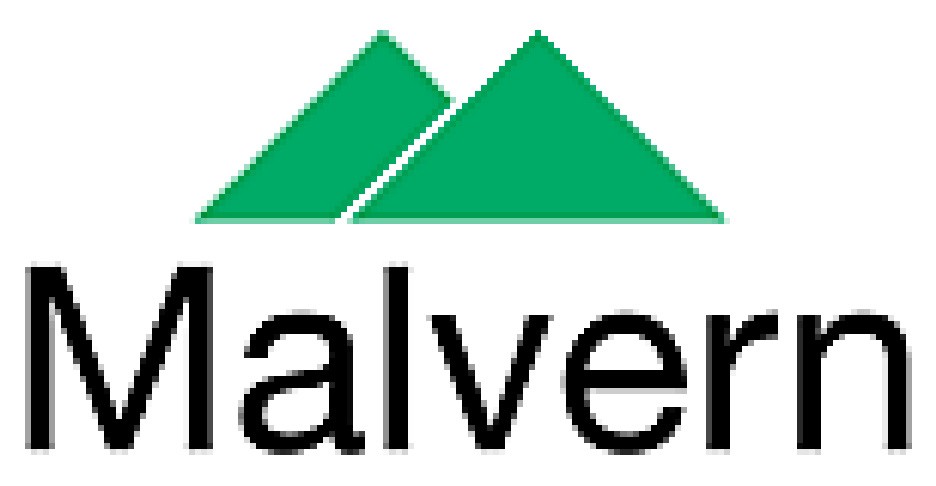
**Size Distribution Report by Number**

*v2.0*

Malvern Instruments Ltd - © Copyright 2008

# Sample Details

| **Sample Name:** | 3-1 1 |  | |
| --- | --- | --- | --- |
| **SOP Name:** | Gonzalo.sop |  |  |
| **General Notes:** |  |  |  |
| **File Name:** | 240717.dts | **Dispersant Name:** | Water |
| **Record Number:** | 15 | **Dispersant RI:** | 1,330 |
| **Material RI:** | 1,59 | **Viscosity (cP):** | 0,8876 |
| **Material Absorbtion:** | 0,01 | **Measurement Date and Time:** | lunes, 24 de julio de 2017... |

**System**

Temperature (°C):

Count Rate (kcps):

25,1

174,3

Duration Used (s):

Measurement Position (mm):

100

4,65

**Cell Description:**

Disposable sizing cuvette

**Attenuator:** 9

## Results

Z-Average (d.nm):

PdI:

Intercept:

# Result quality

Record 15: 3-1 1

Size Distribution by Number

25

20

15

10

5

0

0.1

1

10

100

1000

10000

Size (d.nm)

Number (%)

115,6

0,183

0,946

## Good

Peak 1:

Peak 2:

Peak 3:

Diam. (nm)

64,65

0,000

0,000

% Number

100,0

0,0

0,0

Width (nm)

20,73

0,000

0,000

**Fig 1B**


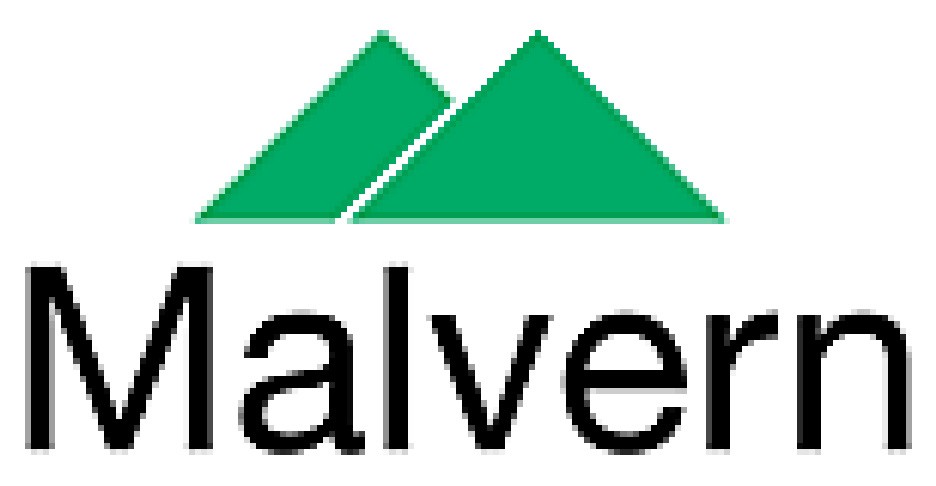
**Size Distribution Report by Number**

*v2.0*

Malvern Instruments Ltd - © Copyright 2008

# Sample Details

| **Sample Name:** | 3-3 2 |  | |
| --- | --- | --- | --- |
| **SOP Name:** | Gonzalo.sop |  |  |
| **General Notes:** |  |  |  |
| **File Name:** | 240717.dts | **Dispersant Name:** | Water |
| **Record Number:** | 20 | **Dispersant RI:** | 1,330 |
| **Material RI:** | 1,59 | **Viscosity (cP):** | 0,8881 |
| **Material Absorbtion:** | 0,01 | **Measurement Date and Time:** | lunes, 24 de julio de 2017... |

**System**

Temperature (°C):

Count Rate (kcps):

25,1

195,9

Duration Used (s):

Measurement Position (mm):

100

4,65

**Cell Description:**

Disposable sizing cuvette

**Attenuator:** 9

## Results

Z-Average (d.nm):

PdI:

Intercept:

# Result quality

Record 20: 3-3 2

Size Distribution by Number

25

20

15

10

5

0

0.1

1

10

100

1000

10000

Size (d.nm)

Number (%)

119,9

0,150

0,949

## Good

Peak 1:

Peak 2:

Peak 3:

Diam. (nm)

81,63

0,000

0,000

% Number

100,0

0,0

0,0

Width (nm)

24,04

0,000

0,000
