# Supplementary material for: Antinociceptive antibiotics-loaded into solid lipid nanoparticles of prolonged release: Measuring pharmacological efficiency and time span on chronic monoarthritis rats
Source: PLoS One. 2018 Apr 12;13(4):e0187473. doi: 10.1371/journal.pone.0187473 (PMC5896893; doi:10.1371/journal.pone.0187473)
Supplement: S2 Fig — Shows the HPLC for the pure antibiotic (A). After completion of one experiment, (minocycline injection, Randall-Selitto test) the CSF was withdrawn and measured by HPLC, (B). (DOCX) [file pone.0187473.s003.docx]

| S A M P L E | | I N F O R M A T I O N | |
| --- | --- | --- | --- |
| Sample Name: | standar M2A | Acquired By: | System |
| Sample Type: | Unknown | Date Acquired: | 06-10-2014 13:22:48 |
| Vial: | 1 | Acq. Method Set: | CarlosValdes |
| Injection #: | 1 | Date Processed: | 09-10-2014 16:31:34 |
| Injection Volume: | 20,00 ul | Processing Method: | prueba |
| Run Time: | 15,0 Minutes | Channel Name: | Wvln Ch1 |
| Sample Set Name: |  | Proc. Chnl. Descr.: | PDA 273,0 nm |

3,00


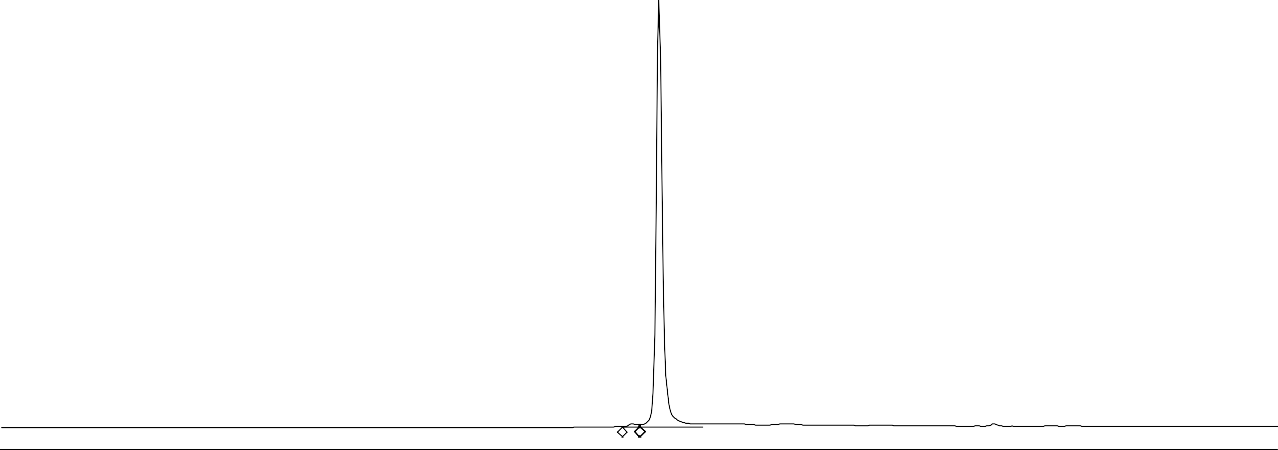

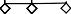


7,718

2,00

AU

1,00

7,419

8,364

8,624

9,214

9,860

11,637

0,00

2,00 4,00 6,00 8,00 10,00 12,00 14,00

Minutes


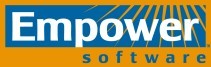


|  | RT | Area | % Area | Height |
| --- | --- | --- | --- | --- |
| 1 | 7,419 | 206645 | 0,98 | 26425 |
| 2 | 7,718 | 18843833 | 89,72 | 3486531 |
| 3 | 8,364 | 458866 | 2,18 | 27286 |
| 4 | 8,624 | 449332 | 2,14 | 23314 |
| 5 | 9,214 | 724139 | 3,45 | 24515 |
| 6 | 9,860 | 193337 | 0,92 | 10675 |
| 7 | 11,637 | 126561 | 0,60 | 21570 |

0,060

# Match Plot

6,00

# Match Plot

0,050

0,040

AU

5,00

4,00

226,1251,9

202,6

270,8

331,3Peak #2

0,030

192,1

Peak #1

250,8

366,9

0,020

0,010

200,00 250,00 300,00 350,00 400,00

nm

3,00

2,00

AU

1,00

200,00 250,00 300,00 350,00 400,00

nm

# Match Plot Match Plot

Peak #4

264,9

351,6

0,08

0,08

0,06

AU

0,06

0,04

0,02

0,04

0,02

AU

0,00

196,8

Peak #3

270,8

354,0

200,00 250,00 300,00 350,00 400,00

nm

0,00

200,00 250,00 300,00 350,00 400,00

nm

# Match Plot Match Plot

Peak #6

244,9

324,2

0,10

0,08

0,06

0,06


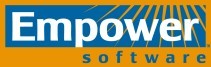


Peak #5

355,2

AU

0,04

0,02

0,00

200,00 250,00 300,00 350,00 400,00

nm

0,04

0,02

AU

0,00

200,00 250,00 300,00 350,00 400,00

nm

## Reported by User: System Project Name: ClaudioLaurido

0,06

# Match Plot


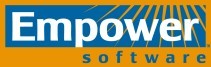


0,04

209,6

Peak #7

261,4

374,1

AU

0,02

0,00

200,00 250,00 300,00 350,00 400,00

nm

# Auto-Scaled Chromatogram

0,00


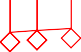

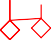

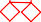


7,419

7,718

8,364

8,624

9,214

9,860

11,637

-0,05

-0,10

AU

-0,15

2,00 4,00 6,00 8,00 10,00 12,00 14,00

Minutes


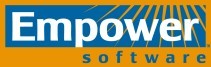


| S A M P L E | | I N F O R M A T I O N | |
| --- | --- | --- | --- |
| Sample Name: | Lcr M1 | Acquired By: | System |
| Sample Type: | Unknown | Date Acquired: | 06-10-2014 13:47:19 |
| Vial: | 1 | Acq. Method Set: | CarlosValdes |
| Injection #: | 1 | Date Processed: | 09-10-2014 16:32:05 |
| Injection Volume: | 20,00 ul | Processing Method: | prueba |
| Run Time: | 15,0 Minutes | Channel Name: | Wvln Ch1 |
| Sample Set Name: |  | Proc. Chnl. Descr.: | PDA 273,0 nm |

0,14


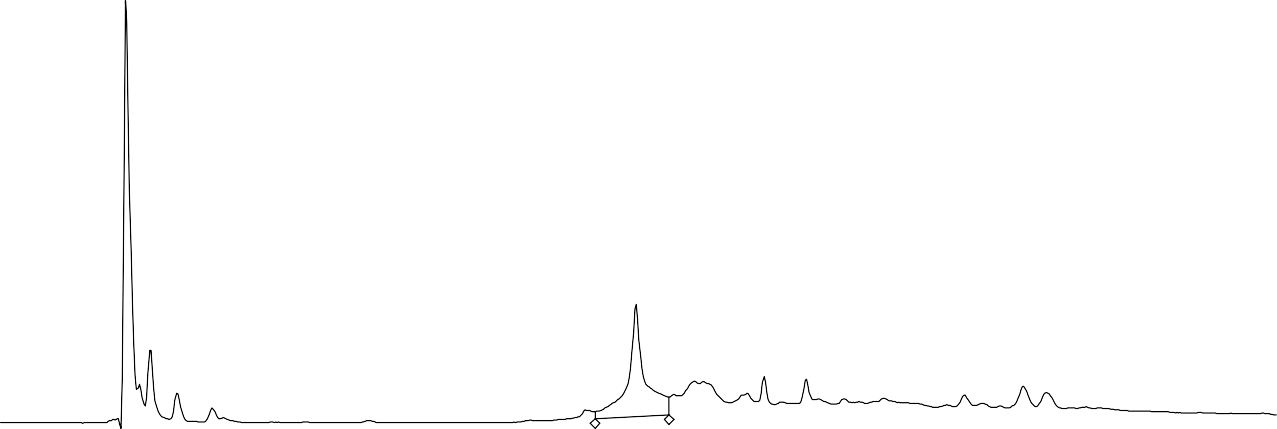


0,12

0,10

0,08

AU

0,06

7,497

0,04

0,02

0,00

2,00 4,00 6,00 8,00 10,00 12,00 14,00

Minutes

|  | RT | Area | % Area | Height |
| --- | --- | --- | --- | --- |
| 1 | 7,497 | 543463 | 100,00 | 38939 |

0,10

**Match Plot**

0,08

190,9

Peak #1

268,5

354,0

0,06

AU

0,04

0,02

0,00

200,00 250,00 300,00 350,00 400,00

nm

0,050

**Auto-Scaled Chromatogram**


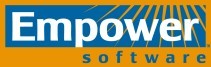


0,040


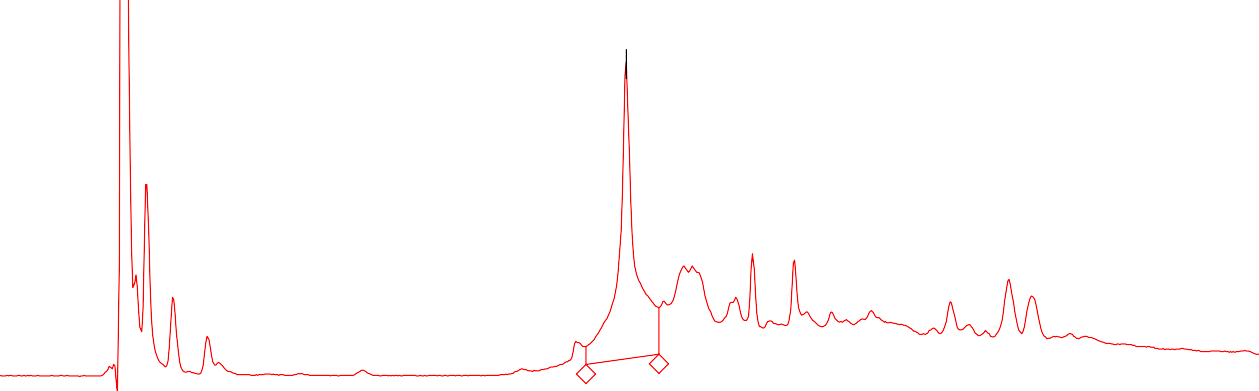


7,497

0,030

0,020

AU

0,010

0,000

2,00 4,00 6,00 8,00 10,00 12,00 14,00

Minutes
