# Supplementary material for: Antinociceptive antibiotics-loaded into solid lipid nanoparticles of prolonged release: Measuring pharmacological efficiency and time span on chronic monoarthritis rats
Source: PLoS One. 2018 Apr 12;13(4):e0187473. doi: 10.1371/journal.pone.0187473 (PMC5896893; doi:10.1371/journal.pone.0187473)
Supplement: S3 Fig — Shows the HPLC for ciprofloxacin for the pure antibiotic (A). After completion of one experiment, (minocycline injection, Randall-Selitto test) the CSF was withdrawn and measured by HPLC, (B). (DOCX) [file pone.0187473.s004.docx]

| S A M P L E | | I N F O R M A T I O N | |
| --- | --- | --- | --- |
| Sample Name: | Cipro LCR | Acquired By: | System |
| Sample Type: | Unknown | Date Acquired: | 06-11-2014 15:14:59 |
| Vial: | 1 | Acq. Method Set: | CarlosValdes |
| Injection #: | 1 | Date Processed: | 30-12-2014 15:39:08 |
| Injection Volume: | 20,00 ul | Processing Method: | prueba |
| Run Time: | 15,0 Minutes | Channel Name: | Wvln Ch1 |
| Sample Set Name: |  | Proc. Chnl. Descr.: | PDA 273,0 nm |

3,00


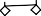


7,131

2,50

2,00

1,50

AU

1,00

0,50

0,00

2,00 4,00 6,00 8,00 10,00 12,00 14,00

Minutes


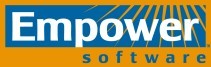


|  | RT | Area | % Area | Height |
| --- | --- | --- | --- | --- |
| 1 | 7,131 | 19136113 | 100,00 | 3354806 |


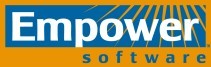


**Match Plot**

6,00

287,4

Peak #1

206,1

315,9

5,00

4,00

3,00

AU

2,00

1,00

0,00

200,00 250,00 300,00 350,00 400,00

nm


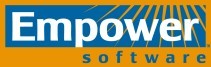


| S A M P L E | | I N F O R M A T I O N | |
| --- | --- | --- | --- |
| Sample Name: | Cipro LCR | Acquired By: | System |
| Sample Type: | Unknown | Date Acquired: | 06-11-2014 15:44:49 |
| Vial: | 1 | Acq. Method Set: | CarlosValdes |
| Injection #: | 1 | Date Processed: | 30-12-2014 15:38:49 |
| Injection Volume: | 20,00 ul | Processing Method: | prueba |
| Run Time: | 15,0 Minutes | Channel Name: | Wvln Ch1 |
| Sample Set Name: |  | Proc. Chnl. Descr.: | PDA 273,0 nm |

0,10


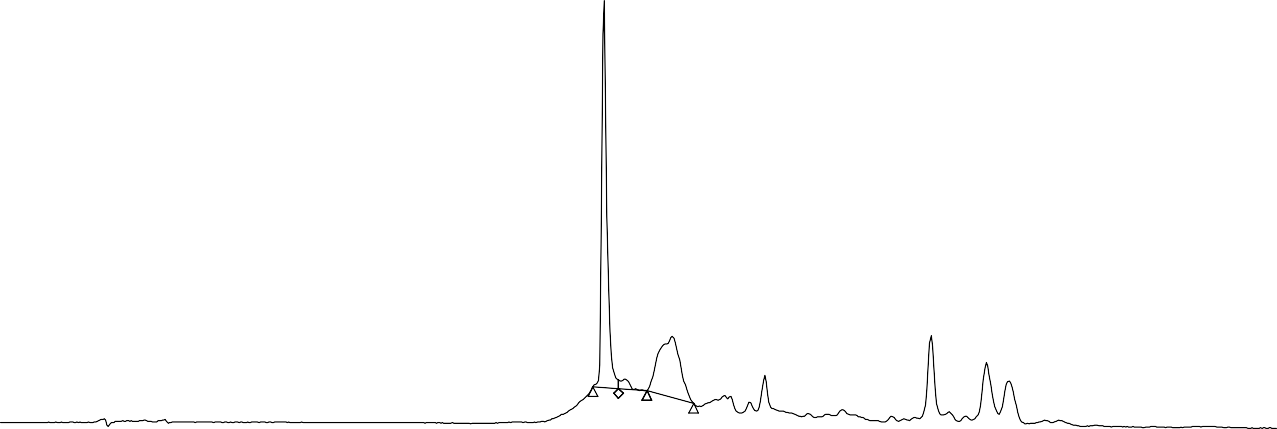


7,116

0,08

0,06

AU

0,04

7,358

7,914

0,02

0,00

2,00 4,00 6,00 8,00 10,00 12,00 14,00

Minutes

|  | RT | Area | % Area | Height |
| --- | --- | --- | --- | --- |
| 1 | 7,116 | 381010 | 58,83 | 88999 |
| 2 | 7,358 | 17699 | 2,73 | 2273 |
| 3 | 7,914 | 248918 | 38,44 | 14401 |

0,10

**Match Plot**

0,025

**Match Plot**

203,8

Peak #2

260,2

326,6

313,5348,0374,1

0,08

0,020

0,06

203,8

279,1

Peak #1

317,1

AU

0,04

0,02

0,00

200,00 250,00 300,00 350,00 400,00

nm

0,015

0,010

AU

0,005

0,000

200,00 250,00 300,00 350,00 400,00

nm

**Match Plot**

0,08

193,3

Peak #3

215,5

268,5

0,06

0,04

AU

0,02

0,00

200,00 250,00 300,00 350,00 400,00

nm

0,015

**Auto-Scaled Chromatogram**


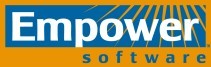


0,010


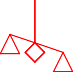


7,116

7,358

7,914

0,005

AU

0,000

-0,005

2,00 4,00 6,00 8,00 10,00 12,00 14,00

Minutes
