# Supplementary material for: Antinociceptive antibiotics-loaded into solid lipid nanoparticles of prolonged release: Measuring pharmacological efficiency and time span on chronic monoarthritis rats
Source: PLoS One. 2018 Apr 12;13(4):e0187473. doi: 10.1371/journal.pone.0187473 (PMC5896893; doi:10.1371/journal.pone.0187473)
Supplement: S4 Fig — The nanoparticles appear rounded, sometimes aggregated in two, but never agglomerated (A). Shows SEM pictures of two representative empty nanoparticles. Image of the smaller nanoparticles found, and the biggest empty nanoparticles found (B). (DOCX) [file pone.0187473.s005.docx]

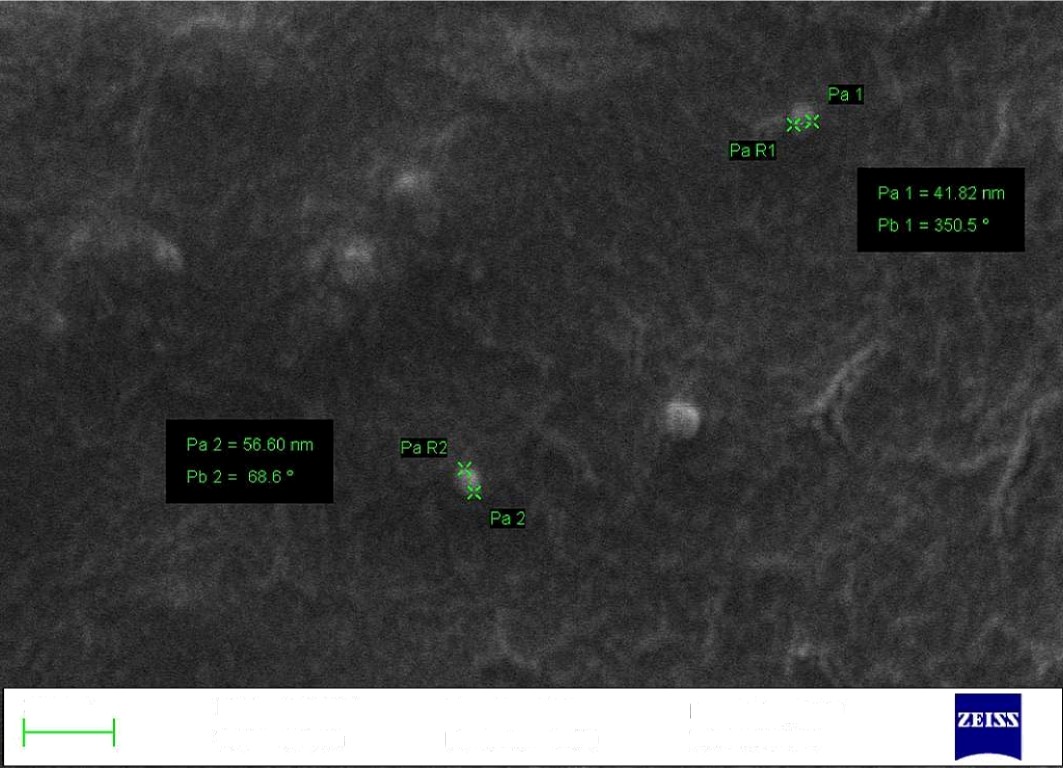


200 nm

EHT = 15.00 kV

\f\/D = 8.O mm

Signal A = SE1

Photo No. = 8590

Date :8 Aug 2017

Time :12:04:07

Fig 4A


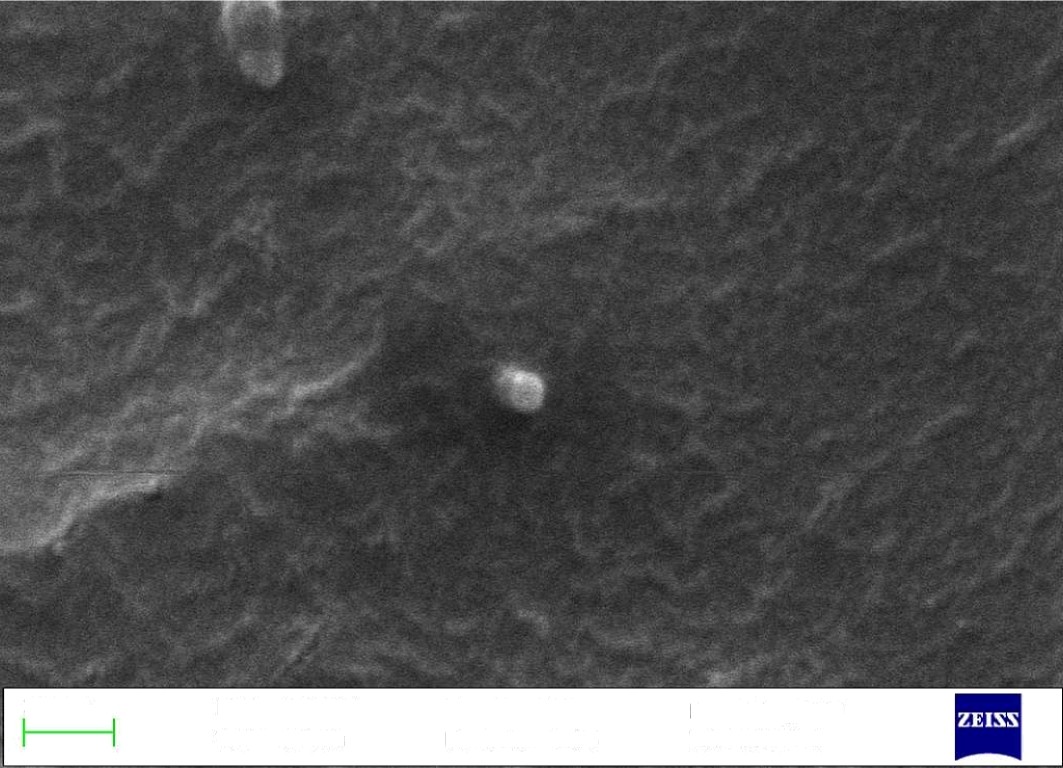


200 nm

EHT = 15.00 kV

\f\/D = 8.O mm

Signal A = SE1

Photo No. = 8593

Date :8 Aug 2017

Time :12:07:55

Fig 4B
